# Environmental surveillance of the health risk of PM_2.5_-bound metals and metalloids in Wuxi, China, from 2020 to 2023

**DOI:** 10.3389/fpubh.2025.1599702

**Published:** 2025-09-08

**Authors:** Lijun Chen, Xuhui Zhang, Xiaoxin Zhou, Yan Gong, Lingcan Kong, Yukang Wu, Wenwei Liu, Pengfei Zhu

**Affiliations:** ^1^The Affiliated Wuxi Center for Disease Control and Prevention of Nanjing Medical University, Wuxi Center for Disease Control and Prevention, Wuxi, China; ^2^Research Base for Environment and Health in Wuxi, Chinese Center for Disease Control and Prevention, Wuxi, China

**Keywords:** air pollution, PM_2.5_, metal, source apportionment, health risk

## Abstract

PM_2.5_ has been a major public concern due to its association with various diseases; however, its contamination is still not well controlled. From 2020 to 2023, the pollution characteristics of PM_2.5_-bound metals and metalloids were monitored in Wuxi, China. The surveillance targeted 26 components, including antimony (Sb), aluminum (Al), arsenic (As), beryllium (Be), cadmium (Cd), chromium (Cr), mercury (Hg), lead (Pb), manganese (Mn), nickel (Ni), selenium (Se), thallium (Tl), barium (Ba), cobalt (Co), copper (Cu), iron (Fe), molybdenum (Mo), silver (Ag), thorium (Th), vanadium (V), zinc (Zn), strontium (Sr), tin (Sn), lithium (Li), uranium (U), and rubidium (Rb). During the study period, The PM_2.5_ mass concentration ranged from 5.00 to 166.0 μg/m^3^, and the annual average PM_2.5_ concentration was 40.4 ± 26.1 μg/m^3^. The total concentration of 22 elements was 659.7 ± 318.5 ng/m^3^. Fe, Al, Zn, Mn, Pb, Cu, and Ba were seven dominant metals in PM_2.5_ accounted for 95.7% of the total metal concentrations (TMs). Both PM_2.5_ and most PM_2.5_-bound metals and metalloids exhibited decreasing trends to varying degrees and seasonal characteristics, peaking in winter. The result of enrichment factor (EF) suggested most elements mainly derived from anthropogenic pollution, while industrial emissions (32.4%), automotive emissions (27.9%), fuel combustion (26.2%) and dust emissions (13.5%) identified as the main sources by the positive matrix factorization (PMF). The hazard quotients (HQs) of all the metals were below 1, with Mn exhibiting highest HQ at 6.29 × 10^−1^ ± 3.28 × 10^−1^. The carcinogenic risks of five elements were as follows: Cd (5.21 × 10^−7^ ± 4.02 × 10^−7^), As (7.00 × 10^−6^ ± 3.83 × 10^−6^), Pb (1.24 × 10^−7^ ± 7.79 × 10^−8^), Ni (3.21 × 10^−7^ ± 1.62 × 10^−7^) and Cr (VI) (2.76 × 10^−6^ ± 1.31 × 10^−6^). These results indicate that both the non-carcinogenic and carcinogenic risks of individual elements monitored were within an acceptable range. However, considerable attention should be given to the comprehensive exposure risk associated with long-term exposure to Mn, As and Cr (VI). This study updated air pollution data, analyzed pollution sources and characteristics and discussed the potential risks of PM_2.5_-bound metals and metalloids. It is of great significance to reduce PM_2.5_ emissions and formulate environmental protection policies to protect the health of local residents.

## Introduction

Particulate matter (PM) pollution is a significant issue in atmospheric environmental governance, negatively impacting air quality, visibility, climate change, atmospheric radiation, and human health (1). In recent years, it has garnered widespread attention in the field of atmospheric science (2–4). PM_2.5_, defined as particulate matter with an aerodynamic equivalent diameter of 2.5 μm or less, has a larger specific surface area, allowing it to effectively accumulate organic compounds, viruses, bacteria, metals and metalloids (5). Over the past decades, a significant amount of epidemiological evidence has indicated PM_2.5_ is associated with diseases. There is a positive correlation between short-term exposure to PM_2.5_ and cardiovascular and respiratory diseases (6), while long-term exposure is significantly correlated with increased mortality from diabetes, cardiovascular disease, lung cancer, and chronic obstructive pulmonary disease (7–10). A study from Thailand found that if PM_2.5_ concentrations reached the World Health Organization’s (WHO) short-term gold standard of 15 μg/m^3^, approximately 8 premature deaths per 100,000 people could be prevented. Moreover, if PM_2.5_ reached the WHO’s long-term gold standard of 5 μg/m^3^, an estimated 159 premature deaths per 100,000 population could be avoided (11). Furthermore, an *in vivo* experiment in mice confirmed that lung metabolite levels were perturbed after PM_2.5_ intratracheal instillation, mainly affecting metabolic pathways such as citric the acid cycle, pyruvate metabolism, purine and pyrimidine metabolism, as well as valine, leucine, and isoleucine metabolism.

In addition, PM_2.5_ exposure affected the relative abundance of *Ruminococcaceae*, *Enterobacteriaceae,* and *Pseudomonas* (12). Moreover, Tong-Hong Wang et al. (13) found that short-term (24-h) exposure to PM_2.5_ could activate the EGFR pathway in lung cancer cells, while long-term (90-day) exposure promoted tumor progression through the activation of EGFR and AhR, by enhancing the TMPRSS2-IL18 pathway. Metals and metalloids, as the important components of PM_2.5_, through inhalation, skin exposure, and oral exposure. They are widespread public concern due to their high toxicity, persistence, bioaccumulation and potential health risks to ecosystems and humans (14, 15). Research has shown that Long-term exposure to high concentrations of metals and metalloids may lead to cardiotoxicity, neurotoxicity, immunotoxicity, and cancer, thereby increasing mortality rates (16, 17).

There is evidence suggesting a close relationship between metal accumulation (such as Zn, Mn, Cu and Ni), abnormal protein expression, and the pathogenesis of neurodegenerative diseases (18). Additionally, Baoman Li et al. (19) found that excessive intake of metals and metalloids (Such as Hg, Pb, As, Al, Bi, Zn, Cu, Cd, Ni, Mn, and Fe) could disrupt the homeostasis and neuroprotective functions of astrocytes, including the glutamate/GABA-glutamine shuttle, antioxidant mechanisms, and energy metabolism, ultimately leading to neuronal damage and initiating neurodegenerative diseases. The skeletons serve as long-term storage site for lead and other metals, accounting for approximately 90% of the total body burden in mammals. Studies have shown that metal exposure (especially as Hg, Pb, Cd, Cr, Al, and Ni) increases the risk of osteoporosis and fractures (20). Moreover, toxic metal ions could exert harmful effects by strongly interacting with essential biomolecules, replacing vital metal ions in proteins, enzymes, or hard structures such as bones and teeth. Additionally, metals with redox properties are key inducers of reactive oxygen species, which may lead to oxidative stress and cellular damage (21).

Although metals and metalloids account for less than 10% of PM_2.5_, it is crucial to analyze their pollution characteristics and the impacts on human health due to their toxicity and enrichment effects. In recent years, many scholars have focused on assessing the health risks of metals in PM_2.5._ Data from Kolkata illustrated that concentrations of carcinogenic metals, such as Ni, Pb, Cd, and Cr (VI), exceeded the guideline limits set by U. S. Environmental Protection Agency (USEPA) (22). A study conducted in a smelting district in Northeast China found that Cd and Pb in PM_2.5_ posed non-carcinogenic health risks, while Cd also presented a potential carcinogenic risk to human health (23).

Literature showed that PM_10_ may originate from resuspended soil and road dust, construction, crystalline sea salt, etc. PM_0.1_ and PM_2.5_ are direct emissions from human activities such as open burning, fuel burning, wildfire smoke, as well as secondary formation of volatile organic compounds, nitrogen oxides, and sulfur oxide pollutants (24). Phairuang et al. (25) found that the primary sources of PM_0.1_ were derived from road traffic, industrial sector, and biomass combustion, in Bangkok, Thailand. The chemical composition and risk assessment of PM_2.5_ were investigated in the brick kiln site and roadside by Ahmad et al. results indicated the combustion sources were the main source of PM_2.5_ (26). In order to reduce the health risks caused by PM2.5, it is necessary to monitor the pollution situation, assess potential release sources and risks, as to take measures to control pollution emissions and protect the health of the population.

Wuxi, a water-bound city located in the lower reaches of the Yangtze River in China, had a permanent residential population of 7.49 million and a Gross Domestic Product (GDP) of 1.55 trillion RMB by the end of 2023. In terms of per capita GDP, Wuxi ranked third in China in 2023. As an economically and socially developed city, Wuxi faces environmental challenges that have triggered a series of health impacts (27–31). A study demonstrated that As and Sb in the drinking water represented an increasing risk to human health (27). Zhu et al. (28) found that cancer risk from the cumulative cancer risk trihalomethanes (THMs) exposure in the drinking water was 1.26 × 10^−4^ while the non-cancer risk was 2.02 × 10^−1^. Additionally, evidence showed that prenatal exposure increased the risk of preterm birth and reduced gestational age in Wuxi (29). As is well known, differences in climate, geographical conditions, socio-economic structure, and lifestyle can result in varying degrees of environmental pollution. Therefore, long-term monitoring and analysis of PM_2.5_ in Wuxi are of great significance.

In Wuxi, there have been few studies on the characteristics, sources, and health risk assessments of PM_2.5_, particularly concerning metals and metalloids in the atmosphere. Wu Keqin et al. (32) studied metals in PM_2.5_ from 2016 to 2020, but their study had limited elements and neglected the sources of metal elements. This article comprehensively monitors the pollution status of PM_2.5_, analyzing the sources and potential health risks of 26 metals and metalloids in PM_2.5_ from 2020 to 2023, including Sb, Al, As, Be, Cd, Cr, Hg, Pb, Mn, Ni, Se, Tl, Ba, Co, Cu, Fe, Mo, Ag, Th, V, Zn, Sr., Sn, Li, U, and Rb. The aim is to provides strategies for air pollution prevention and the protection of public health.

## Materials and methods

### Sample collection

The sampling site was located on the roof of a primary school in Wuxi City, away from the main roads and without nearby pollution sources. The flow rate of the sampler was 100 L/min. Sampling was conducted from January 2020 to December 2023, with samples collected from the 10th to the 16th of each month, or during hazy weather conditions (AQI > 200). In cases of severe pollution, two filter membranes were employed per day, and the samples were stored at −20°C for subsequent analysis. Meteorological parameters, including atmospheric pressure, temperature, relative humidity, precipitation, and wind speed, were recorded at the time of sample collection.

### Sample preparation and detection

The PM_2.5_ samples were pretreated in accordance with the guidelines outlined in the Handbook for Monitoring and Protecting the Health Effects of Air Pollution on People, released by the China Center for Disease Control and Prevention. The filters membranes were accurately weighed, placed into 15 mL centrifuge tubes, and 10 mL of a 5% nitric acid solution was added. The samples were immersed in a 70°C-water bath and subjected to ultrasonication for 3 h. Following this, the samples were thoroughly shaken and centrifuged at 4500 rpm for 5 min and filtered through a 0.45 μm membrane. The concentrations of 26 metals and metalloids were determined using inductively coupled plasma tandem mass spectrometry (ICP-MS/MS, Agilent Technologies 8,900). Before analysis, Quality calibration and resolution verification on the tuning solution of the mass spectrometer was performed. The tuning solution of the mass spectrometer should be measured at least 4 times, and the relative standard deviation of the signal intensity of the elements contained in the measured tuning solution should be confirmed to be ≤ 5%. Under optimized instrument conditions, the reagent blank, standard curve series, sample solution, and quality control sample solution are measured sequentially. The results showed that the concentration measurements of calibration blank, reagent blank, and filter membrane blank for all elements were below the detection limit. The results of certified reference materials are within the scope of the certificate and controllable. The relative standard deviation of the parallel measurement results for all detected elements is less than 8.5%. Then the element recovery percentage from the standard reference material was between 85 and 113%.

### Enrichment factor

The enrichment factor (EF), a unitless index was used to differentiate the sources of metals and metalloids in PM_2.5_ in Wuxi City. EF values greater than 10 (EF > 10) indicate pollution from anthropogenic sources, values less than or equal to 1(EF ≤ 1) indicate natural sources, and values between 1 and 10 (1 < EF ≤ 10) imply a combination of both sources (33, 34). In this study, Al, an element due to its high concentration, good stability in soil, and widespread presence in PM_2.5_, was selected as the reference element. The EF was calculated by applying the following equation (Equation 1).


(1) $${\mathrm{EF}}_{i}=\frac{\left(\frac{C_{i}}{C_{\mathrm{Al}}}\right)}{\left(\frac{B_{i}}{B_{\mathrm{Al}}}\right)}$$

Where Ci represents the concentration of each heavy metal in PM_2.5_ (ng/m^3^), C_Al_ represents the concentration of Al in PM_2.5_ (ng/m^3^), Bi represents the concentration of each heavy metal in the soil (mg/kg), and B_Al_ represents the concentration of Al in the soil. In this study, the background values of soil elements were collected from Nanjing, China (35).

### PMF analysis

PMF is widely used for source apportionment of atmospheric particulate matter (36). This model is a multivariate factor analysis method that identifies and quantifies pollution sources by analyzing the temporal variation in different components. The underlying principle of the PMF can be found in the literature (37). In this research, source apportionment of PM2.5-bound elements in the atmosphere of Wuxi was conducted using PMF 5.0. The calculation of data uncertainty (Unc) is as follows:


(2) $$\mathrm{Unc}=\frac{5}{6}\times \mathrm{MDL}$$

(3) $$\mathrm{Unc}=\sqrt{{\left(\mathrm{Er}\times \text{Conc}\right)}^{2}+{\left(0.5\times \mathrm{MDL}\right)}^{2}}$$

Where Unc represents the uncertainty of the target pollutant, MDL represents the detection limit of the target pollutant, Conc represents the concentration of the target pollutant, and Er represents the error fraction. If Conc ≤ MDL, Unc is calculated using Equation 2; if Conc > MDL, Unc is calculated using Equation 3. In this study, Er was set to 10%.

### Health risk assessment

This study employed the ‘four-step’ health risk assessment model recommended by the U. S. Environmental Protection Agency (EPA) to evaluate the non-carcinogenic and carcinogenic risks of metals and metalloids in PM_2.5_ in Wuxi City. The ‘four-step’ health risk assessment model includes hazard identification, dose–response assessment, exposure assessment, and risk characterization. The toxicological parameters of various metals and metalloids were obtained from Agency for Toxic Substances and Disease Registry (ATSDR) (38), The California Environmental Protection Agency (CALEPA) (39), the Integrated Risk Information System (IRIS) (40), the Provisional Peer Reviewed Toxicity Values (PPRTVs) (41), the US EPA’s National Ambient Air Quality Standards (NAAQS), and other related documents (42), as summarized in Supplementary Table S1. The health risk assessment for metals and metalloids entering the human body via inhalation was calculated using Equations 4–7.


(4) $$\text{Average Daily Doses}\;(\mathrm{ADD})=\frac{C\times \mathrm{ED}\times \mathrm{EF}\times \mathrm{ET}}{\mathrm{AT}}$$

(5) $$\text{Lifetime Average Daily Doses}\;(\text{LADD})=\frac{C\times \mathrm{ED}\times \mathrm{EF}\times \mathrm{ET}}{\mathrm{LT}}$$

(6) $$\text{Hazard Quotient}=\frac{\mathrm{ADD}}{\mathrm{RfC}\times 10^{6}}$$

(7) $${\text{RISK}}_{i}=\text{LADD}\times \mathrm{IUR}\times 10^{-3}$$

Where ADD shows the chronic non-carcinogenic daily exposure dose via inhalation (ng/m^3^), and LADD denotes the daily exposure dose to carcinogenic substances via inhalation routes (ng/m^3^). C is the concentration of each element (ng/m^3^), ED is the exposure duration—set at 30 years according to the ATSDR recommendations. EF illustrates the exposure frequency (EF = 365 days/year), and ET is the exposure time (24 h/day). AT is the average exposure time, which, in this study, is 30 years for chronic non-carcinogenic influences, while LT denotes the lifetime exposure period for carcinogenic effects, set at 70 years. HQ is the non-carcinogenic risk of a heavy metal (unitless) (43), RfC is the reference concentration for chronic non-carcinogenic effects through inhalation (mg/m^3^), and IUR is the inhalation unit risk (μg/m^3^)^−1^. Finally, RISK defines the carcinogenic risk (unitless) (32, 34).

Research has shown that if HQ<1 (34), the non-carcinogenic risk is approximately unlikely or negligible. However, If HQ ≥ 1, a potential health risk is defined. Similarly, if RISK < 10^−6^ (34, 44), indicates the carcinogenic risk is negligible. A RISK value between 10^−6^ and 10^−4^ is considered within acceptable range (34, 44), whereas the carcinogenic risk is acceptable, and if the value of RISK> 10^−4^, it indicates that the carcinogenic risk exceeds acceptable risk limits.

### Data analysis

All the analyses were conducted using R version 4.3.2 (R Foundation for Statistical Computing, Vienna, Austria). Concentration values below the detection limit were replaced with half of the detection limit. Element concentrations are expressed as means ± SD or quartiles [median (P25, P75)]. According to the concentration distribution, one-way ANOVA followed by the Scheffe’s test or Kruskal-Wallis H test followed by Nemenyi test were used for comparison between years and seasons. Spearman’s correlation analysis was used to examine the correlation between various metals and PM_2.5_, as well as meteorological parameters. *p* < 0.05 was set as statistically significant in the study. The seasons were defined as follows: spring (March to May), summer (June to August) autumn (September to November), and winter (December to February).

## Results

### Long-term concentration trends and meteorological characteristics in ambient PM_2.5_ and PM_2.5_-bound metals

In this study, the annual levels of PM_2.5_ between 2020 and 2023 were 48.3 ± 33.2, 41.1 ± 25.1, 36.6 ± 23.3, and 35.7 ± 19.3 μg/m^−3^, respectively. As shown in Table 1, the annual average ambient air PM_2.5_ concentration during the monitoring period was 40.4 ± 26.1 μg/m^3^ (n = 337), with PM_2.5_ concentrations ranging from 5.0–166.0 μg/m^3^. According to the references, the annual PM_2.5_ levels in Wuxi in this study exceeded the limits of the WHO guidelines (AQG2021, 5 μg/m^3^) and also surpassed the primary standard concentration limit of the China National Ambient Air Quality Standards (CNAAQS IT-1, 35 μg/m^3^), but remained within the secondary standard concentration limit of the CNAAQS (IT-2, 75 μg/m^3^).

**Table 1:** The annual average ambient air PM_2.5_ (μg/m^3^) and PM_2.5_-bound metals and metalloids concentration (ng/m^3^) from 2020 to 2023.

Concentration	Mean	SD	Min-Max	P50 (P25-P75)
PM_2.5_	40.4	26.1	5.00–166.0	34.0 (24.0–49.0)
Al	111.9	83.9	14.2–601.7	87.0 (56.4–142.1)
Cr	3.75	1.78	0.28–10.9	3.49 (2.44–4.74)
Mn	31.5	16.4	4.93–94.2	29.0 (19.4–38.7)
Ni	2.88	1.45	0.30–9.20	2.69 (1.88–3.70)
As	3.80	2.08	0.62–9.77	3.27 (2.24–4.82)
Se	2.76	1.45	0.59–9.40	2.47 (1.75–3.41)
Cd	0.68	0.52	0.10–3.78	0.52 (0.35–0.87)
Sb	2.11	1.33	0.33–8.65	1.80 (1.22–2.44)
Tl	0.16	0.13	0.02–0.93	0.12 (0.07–0.19)
Pb	24.1	15.2	6.03–97.0	20.3 (14.2–29.1)
Li	0.46	0.30	0.37–3.29	0.37 (0.37–0.37)
V	0.97	0.60	0.04–3.71	0.87 (0.44–1.28)
Cu	13.1	9.76	1.01–71.7	10.3 (7.39–14.5)
Zn	110.3	67.4	9.65–536.0	94.5 (70.0–136.7)
Ba	10.0	7.55	1.49–60.4	8.24 (5.77–12.3)
Co	0.32	0.21	0.03–1.13	0.29 (0.11–0.43)
Rb	0.86	0.51	0.16–3.96	0.74 (0.51–1.08)
Fe	330.4	174.2	37.6–1027.9	287.2 (202.5–421.1)
Sr	2.77	1.81	0.03–10.2	2.25 (1.54–3.50)
Mo	3.42	2.18	0.03–13.2	3.01 (1.87–4.49)
Ag	0.19	0.19	0.01–1.82	0.15 (0.12–0.15)
Sn	3.40	2.20	0.31–15.6	2.92 (1.94–4.19)

When concentration was less than CNAAQS IT-2, it was defined as a clean day. The average PM_2.5_ concentration on clean days was 34.6 ± 15.6 μg/m^3^. During the monitoring period, samples exceeding the CNAAQS IT-2 accounted for 1.85% of the annual days and 8.01% of the total samples. Additionally, 156 samples exceeded the CNAAQS IT-1, accounting for 46.3% of the total samples. Furthermore, we calculated that the number of days exceeding the AQG 2021 was 12 times greater than the number of days exceeding the CNAAQS IT-2.

**Figure 1:**
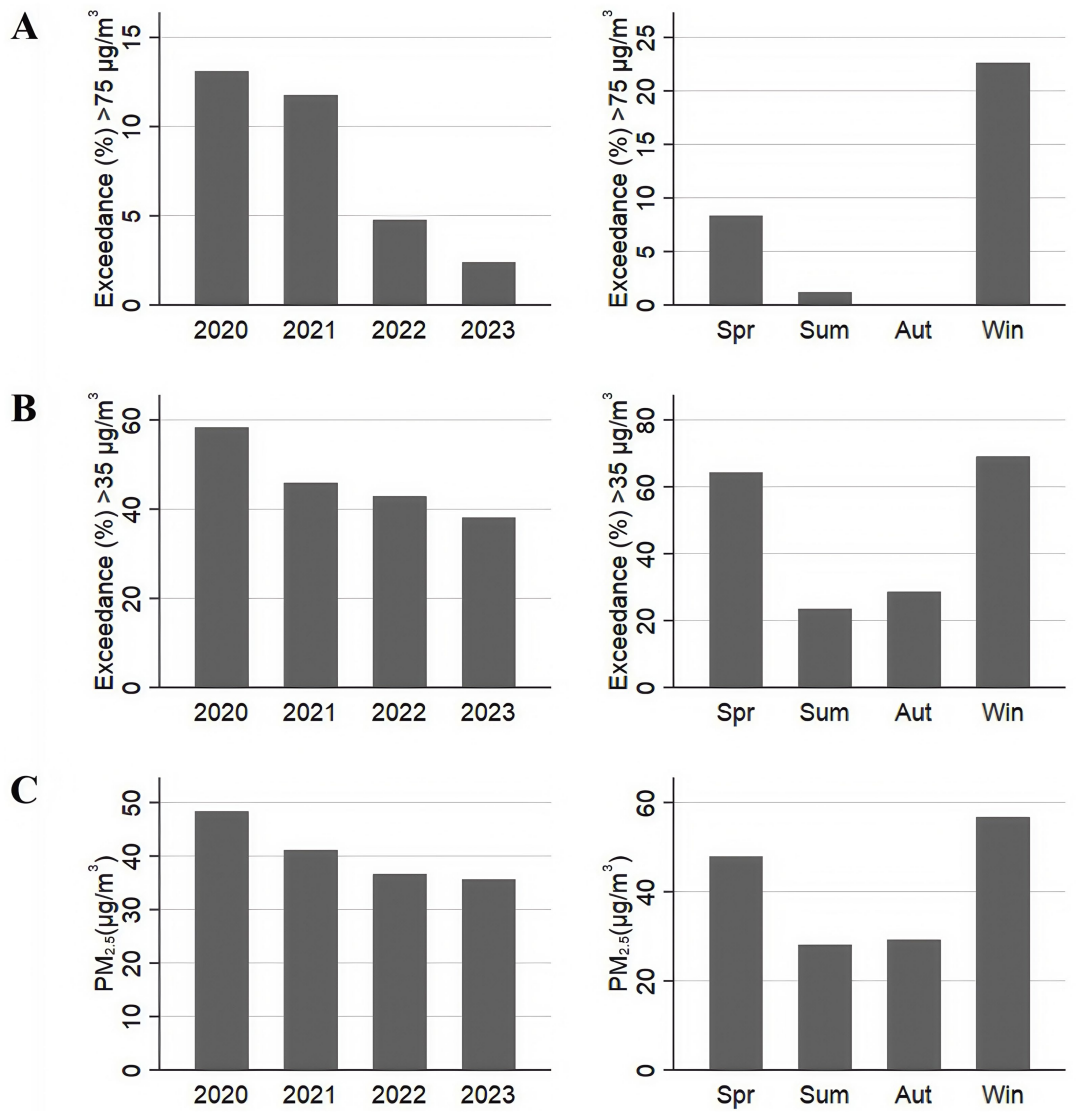
Temporal distribution and exceedance characteristics of PM_2.5_. **(A)** The annual and seasonal distribution characteristics of PM_2.5_ exceeding CNAAQS IT-2 from 2020 to 2023. **(B)** The annual and seasonal distribution characteristics of PM_2.5_ exceeding CNAAQS IT-1 from 2020 to 2023. **(C)** The annual and seasonal distribution characteristics of PM_2.5_ between 2020 and 2023.

Figures 1A,B present the annual and seasonal distribution characteristics of PM_2.5_ exceeding CNAAQS IT-2 and CNAAQS IT-1. According to the CNAAQS IT-2, the proportion of days exceeding the standard to the total monitoring days were 13.10, 11.76, 4.76 and 2.38%, respectively. For CNAAQS IT-1, the exceedance percentages were 58.33, 45.88, 42.86 and 38.10%, respectively, with the percentage of exceeding decreasing each year from 2020 to 2023.

The annual and seasonal distribution characteristics of PM_2.5_ are shown in Figure 1C. Over the last 4 years, the concentration of PM_2.5_ in Wuxi has decreased with increasing years (*p* < 0.01), with a 26% decrease in PM_2.5_ concentration from 2020 to 2023.

We found that the annual average ambient air PM_2.5_ concentration in winter and spring was significantly higher than in summer and autumn (*p* < 0.05). The PM_2.5_ concentration in summer was almost half of that observed in winter. This also confirms that wind speed, temperature, relative humidity, and precipitation were negatively correlated with PM_2.5_ concentration (p < 0.05), while there was a positive correlation between barometric pressure and PM_2.5_ concentration (Table 2).

**Table 2:** The correlation coefficients between PM_2.5_ and PM_2.5_-bound metals and metalloids concentration with meteorological parameters in Wuxi from 2020 to 2023.

Meteorological parameters	AP	T	RH	P	WS
PM_2.5_	0.33*	−0.46*	−0.062	−0.27*	−0.23*
Al	0.30*	−0.31*	−0.29*	−0.37*	−0.054
Cr	0.036	−0.096	0.10	−0.081	−0.41*
Mn	0.31*	−0.36*	−0.032	−0.17*	−0.33*
Ni	0.15*	−0.17*	0.16*	−0.077	−0.26*
As	0.047	−0.11*	−0.11	−0.21*	−0.29*
Se	−0.037	−0.0091	−0.091	−0.28*	−0.42*
Cd	0.16*	−0.22*	−0.22*	−0.27*	−0.15*
Sb	0.12*	−0.21*	−0.21*	−0.37*	−0.39*
Tl	0.22*	−0.31*	−0.26*	−0.33*	−0.16*
Pb	0.42*	−0.56*	−0.11*	−0.22*	−0.22*
Li	0.15*	−0.18*	−0.098	−0.12*	−0.23*
V	0.022	−0.013	−0.0079	−0.21*	0.011
Cu	0.044	−0.11	0.020	−0.15*	−0.38*
Zn	0.33*	−0.34*	0.030	−0.075	−0.30*
Ba	0.44*	−0.49*	−0.11*	−0.24*	−0.13*
Co	0.17*	−0.11	0.094	−0.042	−0.13*
Rb	0.38*	−0.48*	−0.25*	−0.30*	−0.19*
Fe	0.28*	−0.30*	−0.021	−0.16*	−0.30*
Sr	0.34*	−0.31*	−0.17*	−0.30*	−0.079
Mo	0.040	−0.042	0.15*	0.043	−0.43*
Ag	0.10	−0.21*	−0.14*	−0.16*	−0.18*
Sn	−0.061	−0.066	−0.018	−0.15*	−0.26*

AP was atmospheric pressure, T was temperature, RH was relative humidity, P was precipitation and WS was wind speed; * Showed significant correlation at 0.05 level.

During the monitoring period, the total content of 22 elements (except Hg, Th, Be and U, which were below the detection limits) was 659.7 ± 318.5 ng/m^3^, comprising 1.91% mass. The total metal concentrations (TMs) decreased by 30.2%, from 783.4 ng/m^3^ in 2020 to 547.0 ng/m^3^ in 2023. The concentration range of the monitored metals and metalloids was from 0.01 to 1027.9 ng/m^3^. The annual mean concentrations of PM_2.5_-bound metals and metalloids in Wuxi from 2020 to 2023 are shown in Table 1. The mean concentration ranked as Fe > Al > Zn > Mn > Pb > Cu > Ba > As > Cr > Mo > Sn > Ni > Sr. > Se > Sb > V > Rb > Cd > Li > Co > Ag > Tl. The results indicate that the main seven metals and metalloids in PM_2.5_ in Wuxi were Fe, Al, Zn, Mn, Pb, Cu, and Ba. The annual mean concentrations of these seven dominant metals were 330.4 ± 174.2 ng/m^3^, 111.9 ± 83.9 ng/m^3^, 110.3 ± 67.4 ng/m^3^, 31.5 ± 16.4 ng/m^3^, 24.1 ± 15.2 ng/m^3^, 13.0 ± 9.76 ng/m^3^, and 10.0 ± 7.55 ng/m^3^, respectively, which accounted for 95.7% of TMs. The annual mean concentrations of As, Cr, Mo, Sn, Ni, Sr., Se, and Sb were below 10 ng/m^3^ but over 1 ng/m^3^, while the concentrations of remaining PM_2.5-_bound metals and metalloids were less than 1 ng/m^3^.

The annual mean concentrations of As, Cd, Hg and Pb in Wuxi were 3.80 ± 2.08 ng/m^3^, 0.68 ± 0.52 ng/m^3^, 0.1 ng/m^3^ and 24.1 ± 15.2 ng/m^3^, respectively, which are significantly less than the limits set by the CNAAQS (6 ng/m^3^, 5 ng/m^3^, 50 ng/m^3^ and 500 ng/m^3^, respectively).

During the monitoring period over the past 4 years, the concentrations of most metals in PM_2.5_ showed a remarkable decline in Wuxi (*p* < 0.05), as shown in Table 3. Co showed the largest reduction in concentration, dropping by 55.1%, followed by Sr. (47.6%), Cu (39.6%), Fe (33.6%), Rb (33.2%), Tl (32.9%), Zn (31.1%), Cd (30.3%), Al (29.8%), Ba (25.5%), V (21.2%), Se (19.9%), and Mn (17.1%), and others had no statistical trends (*p* > 0.05).

**Table 3:** The annual variations of PM_2.5_-bound metals and metalloids (ng/m^3^) in Wuxi from 2020 to 2023.

Metals and metalloids	2020	2021	2022	2023
Al	135.4 ± 61.8	109.6 ± 109.1	107.4 ± 67.6	95.0 ± 84.8
Cr	3.28 ± 1.58	4.07 ± 1.08	4.26 ± 2.38	3.41 ± 1.70
Mn	34.3 ± 17.2	29.4 ± 16.4	33.8 ± 16.2	28.4 ± 15.2
Ni	2.83 ± 1.16	2.70 ± 1.18	3.50 ± 1.87	2.48 ± 1.29
As	4.18 ± 2.19	3.86 ± 2.31	3.59 ± 1.91	3.57 ± 1.86
Se	3.14 ± 1.76	2.45 ± 1.21	2.94 ± 1.48	2.51 ± 1.20
Cd	0.79 ± 0.52	0.74 ± 0.53	0.63 ± 0.60	0.55 ± 0.37
Sb	2.27 ± 1.34	2.07 ± 1.52	2.07 ± 1.26	2.02 ± 1.17
Tl	0.20 ± 0.13	0.16 ± 0.16	0.12 ± 0.07	0.13 ± 0.12
Pb	25.7 ± 16.3	24.4 ± 15.6	21.6 ± 9.81	24.72 ± 17.7
Li	0.41 ± 0.18	0.47 ± 0.23	0.47 ± 0.34	0.50 ± 0.41
V	1.07 ± 0.58	1.04 ± 0.58	0.92 ± 0.64	0.84 ± 0.59
Cu	16.12 ± 9.90	14.31 ± 10.14	12.0 ± 10.6	9.74 ± 6.86
Zn	129.7 ± 85.9	120.7 ± 62.3	101.3 ± 55.3	89.3 ± 54.8
Ba	12.0 ± 5.77	10.2 ± 4.60	9.08 ± 8.12	8.91 ± 10.2
Co	0.43 ± 0.21	0.45 ± 0.15	0.19 ± 0.14	0.19 ± 0.17
Rb	1.05 ± 0.65	0.82 ± 0.5	0.88 ± 0.45	0.70 ± 0.35
Fe	400.5 ± 198.0	369.8 ± 167.6	284.9 ± 140.9	265.9 ± 150.1
Sr	3.69 ± 1.79	3.23 ± 1.82	2.23 ± 1.48	1.94 ± 1.58
Mo	3.17 ± 2.23	3.47 ± 1.89	4.01 ± 2.24	3.03 ± 2.27
Ag	0.13 ± 0.08	0.20 ± 0.21	0.17 ± 0.09	0.24 ± 0.28
Sn	3.14 ± 1.52	2.88 ± 1.28	4.79 ± 3.08	2.80 ± 1.87

**Figure 2:**
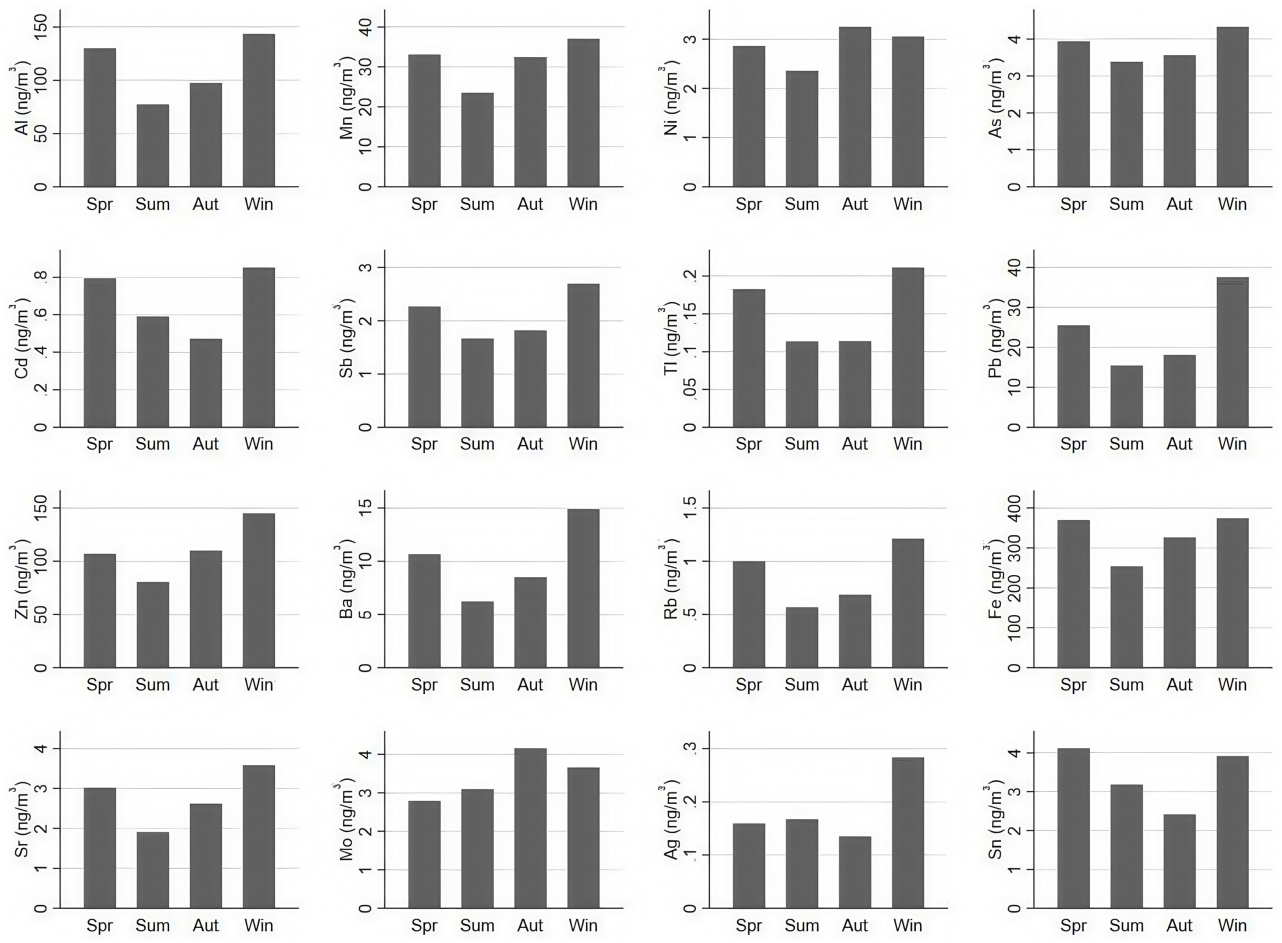
Seasonal distribution characteristics 16 PM_2.5-_bound metals and metalloids in the atmosphere with statistical significance.

The seasonal distribution characteristics of metals and metalloids in PM_2.5_ are shown in Figure 2. The annual average concentrations of Pb, Zn, Ba, Ag, and Rb were significantly higher in winter compared to spring, summer, and autumn (*p* < 0.05). Additionally, the annual average concentrations of Al, Cd, Sb, Tl and Sr. were notably higher in winter than in summer and autumn (*p* < 0.01). The annual average concentrations of Mn, Ni, and As were significantly higher in winter than in summer (*p* < 0.05). Furthermore, the annual average concentrations of Al, Mn, Sb, Tl, Pb, Ba, Rb, Fe, Sr, and Sn were significantly higher in spring than in summer (*p* < 0.05). The annual average concentrations of Cd, Tl, Pb, Rb, Mo, and Sn were significantly higher in spring compared to autumn (*p* < 0.01). Lastly, the annual average concentrations of Mn, Ni, Zn, Fe, and Mo were significantly higher in autumn than in summer (*p* < 0.05), while no significant statistical trends were observed for other elements (*p* > 0.05). Compared to spring, the mean concentration of Al, Mn, Sb, Tl, Pb, Ba, Rb, Fe, Sr. and Sn was 68.4, 41.0, 36.0, 61.3, 65.4, 71.6, 76.7, 45.8, 58.8, and 29.4% higher in summer, respectively. In contrast, the average concentration of Pb, Zn, Ba, Ag, Rb, Al, Cd, Sb, Tl, Sr., Mn, Ni, and As was 143.5, 80.1, 140.0, 69.2, 114.3, 85.6, 44.7, 61.7, 86.7, 88.2, 57.8, 29.4, and 27.9% higher in winter than in summer, respectively.

### Correlation and source analysis of PM_2.5_ and PM_2.5_-bound metals

**Figure 3:**
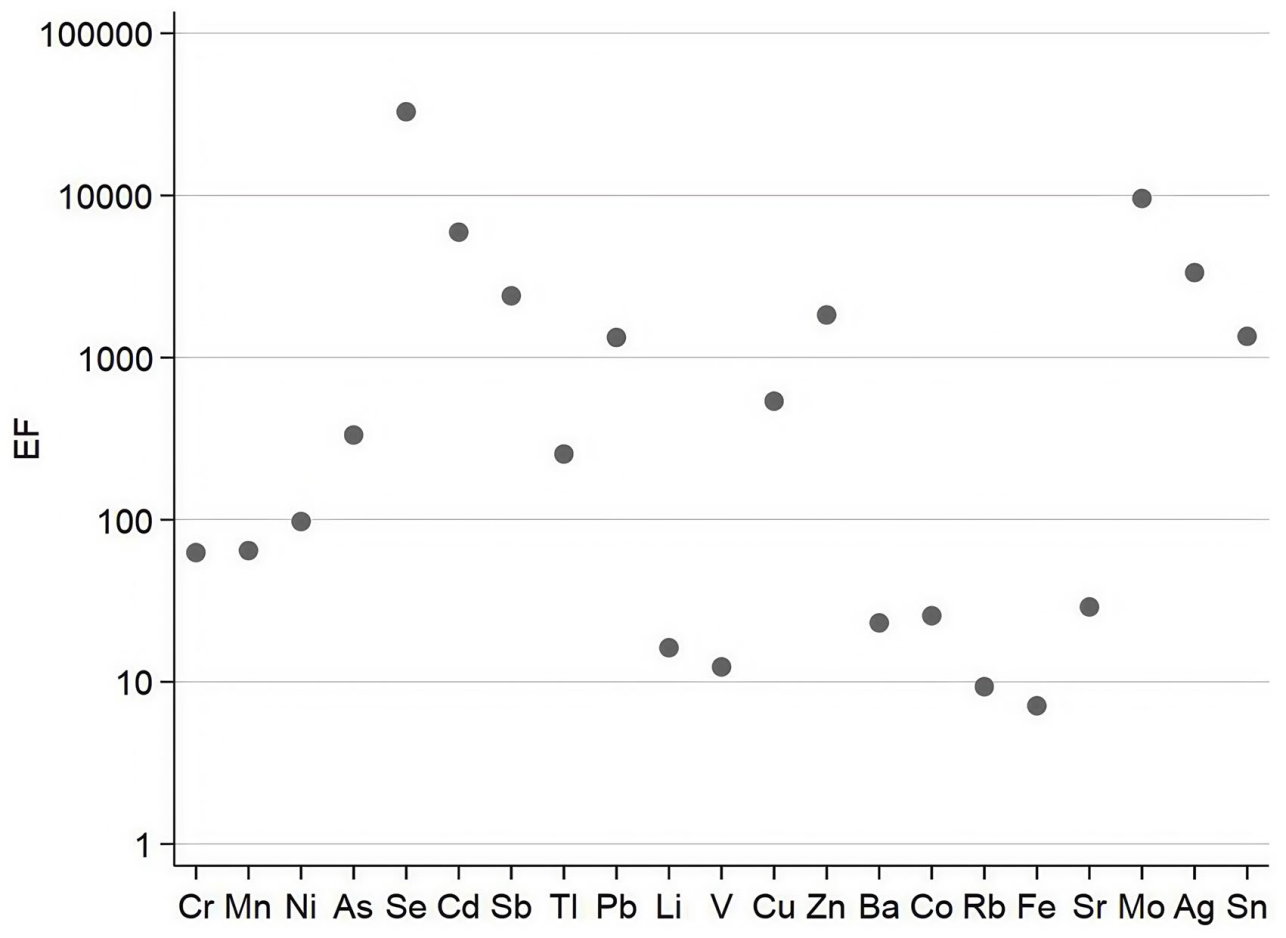
The distribution of EFs for 22 metals and metalloids in the atmosphere during the monitoring period.

The enrichment factor (EF) method is widely used to determine the sources of elements in atmospheric particulate matter, distinguishing between natural and anthropogenic factors. By utilizing the EF method, researchers can evaluate the extent to which human activities—such as industrial emissions and transportation—influence metals in the atmosphere. This assessment is crucial for analyzing air quality and developing environmental policies. The specific distribution of EFs for PM_2.5_-bound metals and metalloids in Wuxi, as showed in this study, is detailed in Figure 3.

Among the 22 monitored metals and metalloids in PM_2.5_, all except Fe, Rb, and the reference element Al displayed EF values higher than 10, indicating significant anthropogenic pollution during the monitoring period. Notably, the EF values for Se, Mo, Cd, Ag, Sb, Zn, Sn, Pb, Cu, As, and Tl exceeded 100, with mean values of 32823.3 ± 20769.2, 9578.7 ± 8559.9, 5938.6 ± 4623.4, 3343.5 ± 4051.8, 2404.6 ± 1628.9, 1833.7 ± 1318.2, 1352.4 ± 1145.7, 1330.1 ± 936.7, 538.7 ± 411.6, 333.2 ± 260.7, and 254.4 ± 179.3, respectively. These significantly high EFs indicate essential anthropogenic pollution and high levels of enrichment. Additionally, the EF values for Ni, Mn, Cr, Sr., Co, Ba, Li, and V ranged between 10 and 100, showing that these metals were mainly influenced by anthropogenic sources and exhibited moderate enrichment. Meanwhile, Rb and Fe had EF values between 1 and 10, indicating contributions from both natural and anthropogenic sources. Since all metals and metalloids analyzed in this study had EF values higher than 1, they need careful consideration. Further studies into their sources and potential risks are needed to better evaluate their effects.

**Figure 4:**
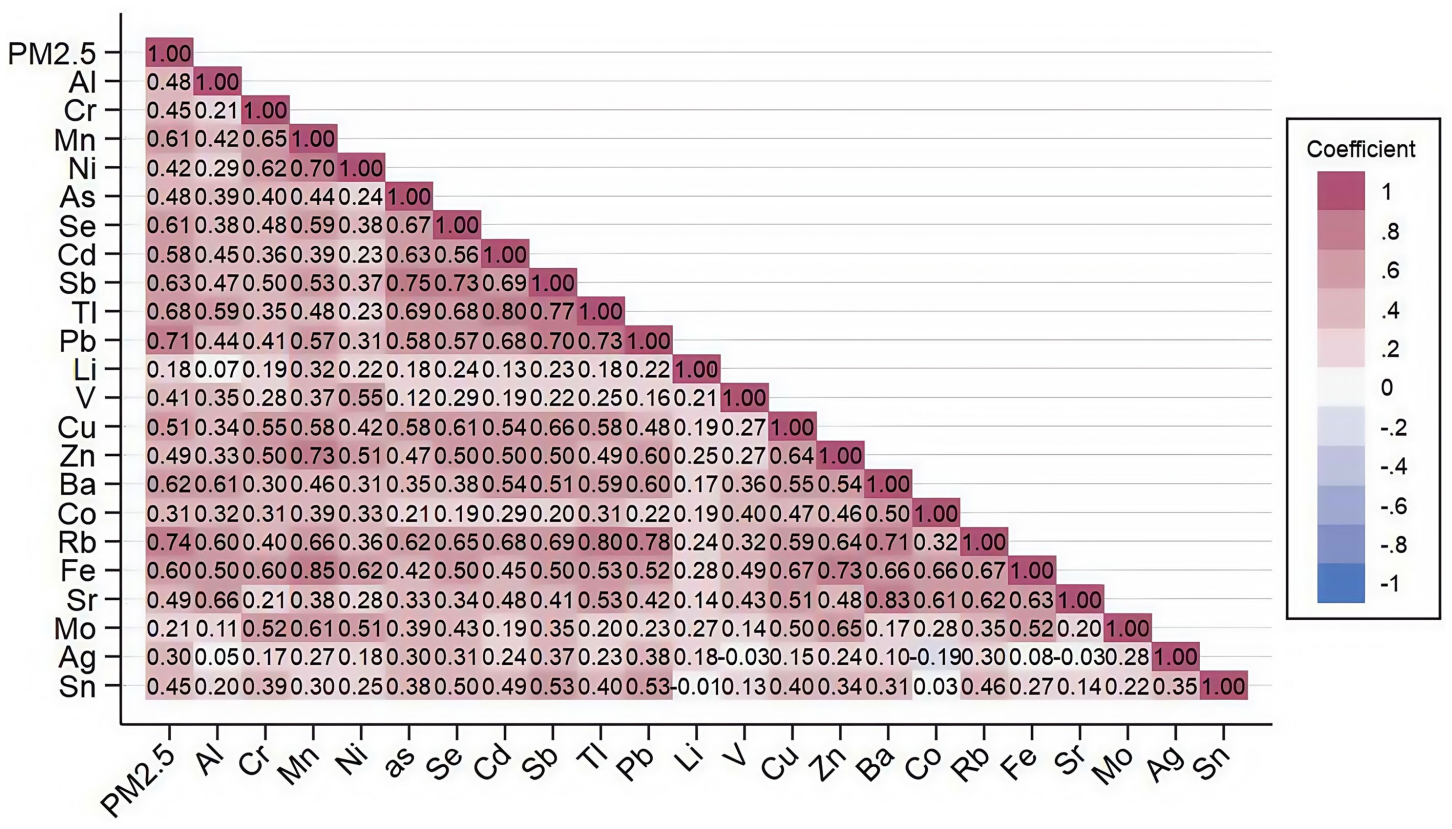
Heat map of correlation coefficient matrix between PM_2.5_ and detected metals and metalloids.

The correlation analysis of PM_2.5_ and PM_2.5_-bound metals and metalloids in Wuxi during the monitoring period is presented in Figure 4. As shown, the annual mean concentrations of the 22 PM_2.5_-bound metals and metalloids exhibited positive correlations with PM_2.5_ concentrations, with the correlation coefficients all higher than 0 (*p* < 0.05). Meanwhile, the correlation coefficients of Mn with Ni, Zn, Fe; Sb with As, Se, Pb; Tl with Cd, Pb, Rb with Sb; Pb with Rb; Zn with Fe; Sr. with Ba were above 0.7 (*p* < 0.05). Additionally, the correlation coefficients of Zn with Cu; Cr with Mn, Ni; Cd with Pb were above 0.6 (*p* < 0.05), indicating strong correlations. In statistics, the stronger the correlation, the greater the likelihood of a common pollution source.

**Figure 5:**
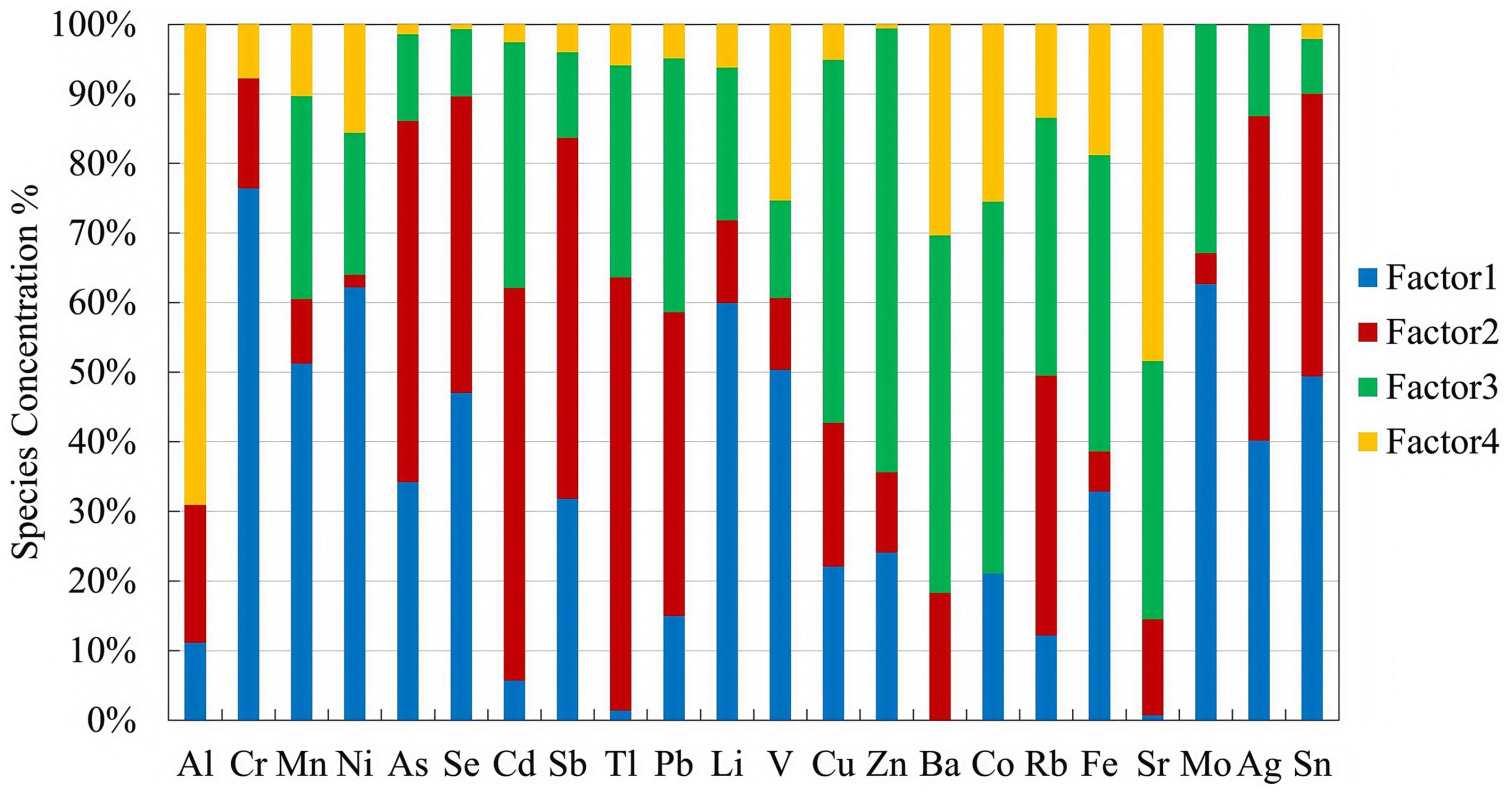
PMF analysis results showing source contributions of PM2.5-bound metals and metalloids in Wuxi.

To investigate the specific sources of metals in PM_2.5_, PMF5.0 was applied to analyze the sources of PM_2.5_-bound metals and metalloids in Wuxi. Four distinct sources were identified by comparing spectral variations across different factors and performing orthogonal matrix rotation operations. As shown in Figure 5, the contribution rates of these four factors were 32.4, 26.2, 27.9, and 13.5%, respectively. The contribution rates of elements such as Cr (76.5%), Mo (62.7%), Ni (62.2%), Li (60%), and Mn (51.3%) in factor 1 were significantly high. Among these, Cr, Mo, Ni, and Mn primarily originate from industries such as automobile manufacturing and electronics production (45). Given the strong presence of the electronic information industry, precision machinery and mechatronics, and automotive parts manufacturing in Wuxi, Factor 1 was identified as an industrial source. The contribution rates of As (51.9%), Se (42.6%), Cd (56.4%), Sb (51.9%), Tl (62.2%), and Pb (43.6%) in Factor 2 were also remarkable. Moreover, strong correlations were observed between As and Se, as well as Cd and Pb—metals commonly associated with fuel combustion (46, 47). Therefore, Factor 2 was identified as a combustion source. In Factor 3, Zn (63.8%), Co (53.4%), and Cu (52.2%), showed high contributions. Zn is linked to motor vehicle exhaust and rubber tire wear, Cu generates from gasoline and diesel vehicle emissions, and Co is primarily used in battery manufacturing for electric vehicles and e-bikes. Hence, factor 3 was identified as a source of automotive emissions. Factor 4 was characterized by a higher rate of Al (69.0%) remarkably exceeding that of other elements. Since Al is the most abundant metal in the Earth’s crust (36) Factor 4 was identified as a natural dust source.

### Health risks

**Figure 6:**
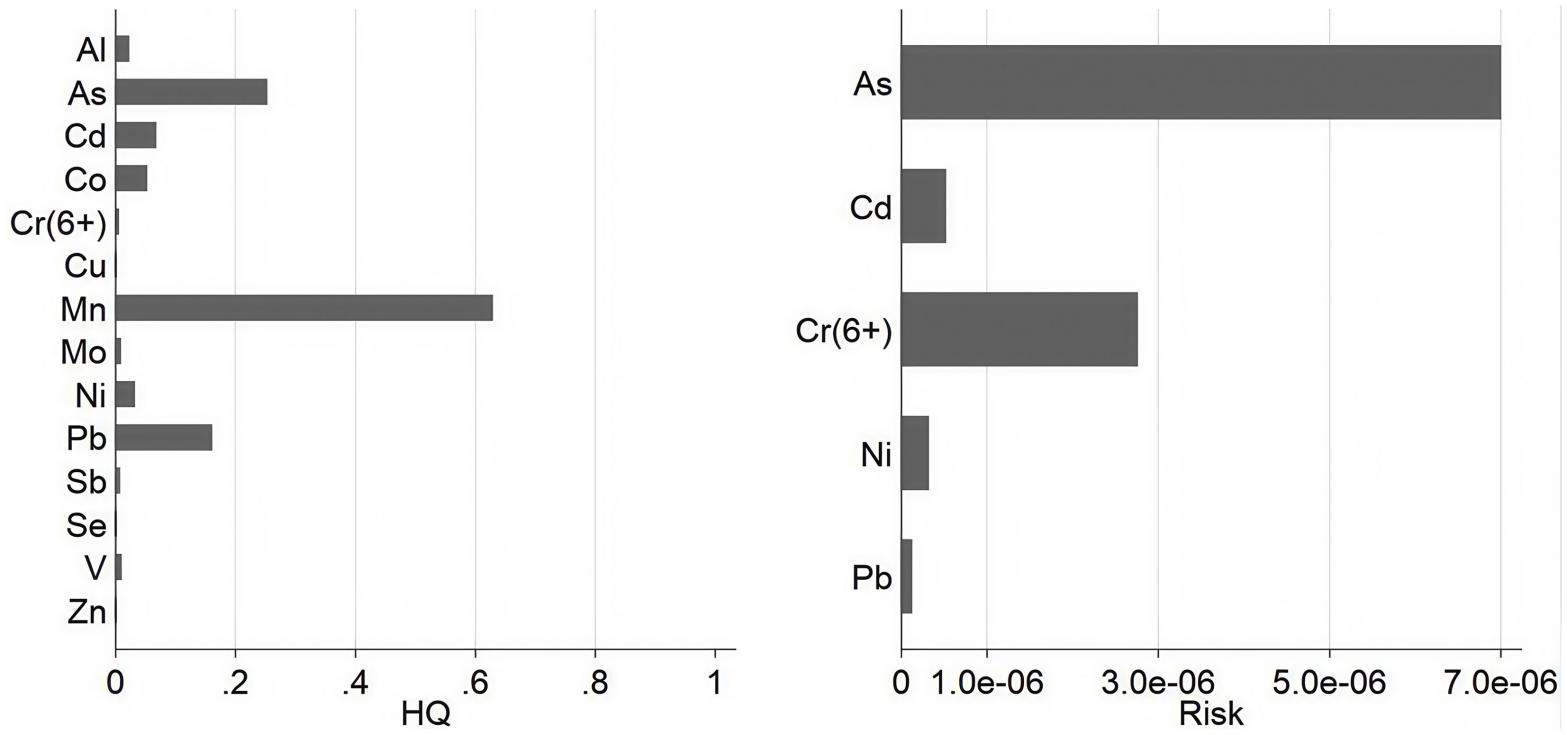
Non-carcinogenic and carcinogenic risks associated with inhalation exposure to PM_2.5_-bound metals and metalloids.

Based on the “four-step” health risk assessment model and the reference toxicity parameters for metals and metalloids, the non-carcinogenic risk for adults exposed to 14 elements in PM_2.5_, and the carcinogenic risk (RISK) for adults exposed to 5 elements in PM_2.5_ via inhalation were calculated. Regarding non-carcinogenic risks (Figure 6), the HQs of Sb, Al, As, Be, Cd, Cr (VI), Hg, Pb, Mn, Ni, Se, Co, Cu, Mo, V and Zn were all less than 1, illustrating no essential non-carcinogenic risk. However, Mn showed the highest non-carcinogenic risk among all other metals and metalloids, with an HQ of 6.29 × 10^−1^ ± 3.28 × 10^−1^, followed by As (2.53 × 10^−1^ ± 1.39 × 10^−1^) and (Pb 1.61 × 10^−1^ ± 1.01 × 10^−1^).

For carcinogenic risks (Figure 6), the RISK values of Cd, Pb, and Ni were all bellow threshold of 1 × 10^−6^ during the monitoring period, with values of 5.21 × 10^−7^ ± 4.02 × 10^−7^, 1.24 × 10^−7^ ± 7.79 × 10^−8^, and 3.21 × 10^−7^ ± 1.62 × 10^−7^, respectively. However, it is important to note that the RISK values of Cr (VI) and As both exceeded 1 × 10^−6^, with values 2.76 × 10^−6^ ± 1.31 × 10^−6^, 7.00 × 10^−6^ ± 3.83 × 10^−6^, respectively, indicating potential carcinogenic concern.

## Discussion

Air pollution has become a major public concern due to its association with various diseases, including lung cancer, cardiovascular disease, bladder cancer, childhood leukemia, dementia, and immune system disorders, all of which can contribute to abnormal death (48–51). According to WHO, approximately 2.4 million people die each year due to the health effects of air pollution (52). Among various pollutants, PM_2.5_ is considered one of the most important environmental risk factors, contributing to cardiovascular disease (53), reduced childhood intelligence (50), asthma (54), chronic obstructive pulmonary disease (55), and allergic disease (56).

During the monitoring period, the concentration of PM_2.5_ in Wuxi showed a significant downward trend, indicating that air pollution control measures have had a certain effect. Additionally, the proportion of clean days relative to the total number of monitoring days increased compared to the standard, suggesting that the emission reduction efforts in Wuxi have been effective over the past 4 years. This enhancement can be attributed to the implementation of “Action Plan for Air Pollution Prevention and Control” and the “Three Year Action Plan for Winning the Blue Sky Defense War.” In recent years, numerous policies have been continuously introduced across China to improve air quality and protect public health, with considerable results. For instance, the annual average concentration of PM_2.5_ in China decreased from 61.8 μg/m^3^ to 42.0 μg/m^3^ in 2017 (57). Similarly, in Suzhou, located southeast China, the average PM_2.5_ concentration declined from 51.2 ± 30.1 μg/m^3^ (2019) to 43.9 ± 25.0 μg/m^3^ (2021) (34). In the Beijing-Tianjin-Hebei (BTH) Region in northern China, the annual average PM_2.5_ concentration decreased from 98.9 μg/m^3^ in 2013 to 64.9 μg/m^3^ in 2017 (58).

However, the annual average concentration of PM_2.5_ in Wuxi was 40.4 ± 26.1 μg/m^3^, which still exceeded both the CNAAQS IT-1 (35 μg/m^3^) and the WHO guideline of 5 μg/m^3^. This indicates that the air pollution remains concerning and requires serious attention.

It was showed that PM_2.5_ concentrations in winter and spring were significantly higher than those in summer and autumn (*p* < 0.05). This seasonal trend is consistent with findings from Suzhou (34) and Hangzhou (59), and may be attributed to lower temperature, reduced precipitation, lower wind speeds and lower relative humidity during winter and spring, resulting in difficult for pollutants to disperse. Therefore, environmental protection departments should focus on atmospheric conditions during these patterns and increase monitoring frequency accordingly.

During the monitoring period, Fe, Al and Zn were identified as the main components of PM2.5-bound metals and metalloids in Wuxi. It is well known that Al and Fe are the third and fourth most common elements in the nature, accounting for approximately 7.73 and 4.75% of its total weight, respectively. In contrast, Zn is mainly associated with tire wear, vehicle exhaust emissions, and the use of Zn in rubber products. This is particularly related in Wuxi, where, as of 2023, there were almost 2.72 million registered vehicles and approximately 105 rubber factories, contributing increasingly to Zn emissions.

The annual mean concentrations of As, Cd, Hg and Pb in Wuxi were significantly lower than the limits set by the CNAAQS. However, it is noteworthy that the annual mean concentrations of total Cr were 3.75 ± 1.78 ng/m^3^. Although previous studies showed that Cr (VI) accounts for about 1/7 of total Cr (60), the estimated concentration of Cr (VI) still exceeded the CNAAQS (0.025 ng/m^3^) by a factor of 20. Cr (VI) is classified as a Class I carcinogen by the International Agency for Research on Cancer (IARC) and enter the human body through ingestion, inhalation or touch with skin and mucous membranes. Therefore, it is necessary to pay special attention to Cr (VI) exposure. In this study Cr (VI) concentration were defined based on literature. Future research should focus on speciation analysis of chromium to provide a more accurate risk assessment associated with Cr (VI).

The concentrations of Co, Sr., Cu, Fe, Rb, Tl, Zn, Cd, Al, Ba, V, Se, and Mn in PM_2.5_ showed a decreasing trend to varying degrees, as analyzed by Scheffe’s test or the Kruskal Wallis H test, as Co showed the largest reduction in concentration, followed by Sr. and Cu This trend was consistent with the decline in PM_2.5_, as positive correlations were observed between PM_2.5_-bound metals and metalloids and PM_2.5_ in Wuxi. This may be due to the implementation of the Action Plan for Air Pollution Prevention and Control and the Three Year Action Plan for Winning the Blue Sky Defense War. Among them, measures such as reducing coal combustion, promoting green travel, and using clean energy have led to a decrease in PM_2.5_ related metal concentrations. The seasonal patterns of metals and metalloids in PM_2.5_ can be attributable to source emissions, meteorological factors, anthropogenic activities, and environmental transport. As shown, the concentrations of PM_2.5_-bound metals and metalloids in Wuxi exhibited obvious seasonality. In addition to meteorological factors, this may be due to the increased use of coal-fired heating in spring and winter, which leads to significant emissions of sulfates and nitrates. These emissions improve the acidity of droplets, increasing the solubility of metal elements and making them more readily captured by particulate matter (61–63).

In this study, the EFs of Se, Mo, Cd, Ag, Sb, Zn, Sn, Pb, Cu, As, and Tl were above 100, indicating that these elements were mainly influenced by anthropogenic sources, exhibiting significant high enrichment and severe pollution, which needs attention. On the other hand, Ni, Mn, Cr, Sr., Co, Ba, Li, and V showed moderate enrichment. Spearman correlation analysis revealed strong correlations between various pairs of metals, such as Mn and Ni, Zn, Fe; Sb and As, Se, Pb; Tl and Cd, Pb, Rb and Sb; Pb and Rb; Zn and Fe; Sr. and Ba; Zn and Cu; Cr and Mn, Ni; Cd and Pb. Based on the EFs and these correlation results, the particular sources of Se, Mo, Cd, Sb, Zn, Pb, Cu, As, Tl, Ni, Mn, Cr, Sr., Co, Ba, and Li needed special attention.

To accurately identify the sources of metal pollutants, the PMF5.0 was utilized in this study. As illustrated in Figure 5, the contribution rates of industrial emissions, automotive emissions, fuel emissions, and dust during the monitoring period in Wuxi were 32.4, 27.9, 26.2, and 13.5%, respectively. According to the Statistical Bulletin on National Economic and Social Development of Wuxi City, from 2020 to 2023, the added value of industrial enterprises above designated size reached 396.9, 492.6, 558.6 and 600.5 billion RMB, increasing by 6.60, 12.9, 5.40 and 7.80% year-on-year, respectively. Industry plays an essential role in Wuxi’s economic and social development. However, as a major source of PM_2.5_-bound metal pollution, it is necessary to optimize the industrial structure and accelerate industrial transformation while maintaining development. The contributions from automobile emissions and combustion suggest the importance of promoting green transportation, clean energy, and transitioning from thermal power to wind and hydropower. Notably, a waste incineration power plant has been built in the east area of Wuxi, which helps control city’s living garbage and provides a cleaner, recyclable energy source compared to conventional electric power generation. Additionally, to control soil dust pollution, attention should be made to enforce civilized construction practices, promote enclosure engineering, harden exposed roads, and wet operations measures.

Regarding non-carcinogenic risks, the HQs of Sb, Al, As, Be, Cd, Cr (VI), Hg, Pb, Mn, Ni, Se, Co, Cu, Mo, V and Zn were all below 1 in Wuxi, indicating that long-term exposure to these metals and metalloids is unlikely to cause adverse health effects. Moreover, the non-carcinogenic risks for residents in Wuxi were lower than other cities in southeastern China, such as Hangzhou and Ningbo (59).

However, it is noteworthy that the risk from inhalation accounts for only a small portion compared to oral and skin exposure routes (64). Therefore, although the non-carcinogenic risk from inhalation remains within an acceptable range, the overall risk of human exposure to various environmental elements should not be overlooked.

As Mn has the highest HQ among all elements in this study, its excessive accumulation in the central nervous system may lead to neurotoxicity, cussing brain disease (65–67). And the disturbance of manganese homeostasis caused by excessive intake of manganese is related to the occurrence of osteoporosis, obesity, type 2 diabetes/insulin resistance, non-alcoholic fatty liver, atherosclerosis and other diseases (68). Co-exposure to manganese, lead, and chromium may exacerbate oxidative stress (69). Al, which had the second highest annual average concentration during monitoring period, has been associated with Alzheimer’s disease, epilepsy and autism (70). As a result, the potential health risks of Mn and Al should not be ignored, and more studies are needed to explore their non-carcinogenic influences on local residents. Notably, the HQs value of Mn in spring and winter compared to summer and autumn, indicating that the non-carcinogenic risks to Wuxi residents posed by Mn in colder seasons need particular attention.

For carcinogenic risks, the RISK values of Cd, Pb, and Ni were below 1 × 10^−6^ for all seasons during the monitoring duration, while the RISK values of Cr (VI) and As were between 1 × 10^−6^ and 1 × 10^−4^. This illustrates that the RISK of Cd, Pb, and Ni via inhalation was negligible.

As the RISK value of Cr (VI) and As were higher than 1 × 10^−6^ for all the seasons. Excessive intake of As will lead to skin cancer, lung cancer, bladder cancer, liver cancer, cardiovascular disease and nervous system damage (71, 72). It was indicated that combined effect of As and Cd were synergistic effect, and Cd may potentiate As nephrotoxicity during the long-term (73). Cr (VI) is related with carcinogenic, genotoxic, and mutagenic effects (74, 75). Chronic exposure to Ni, Cr (VI) or As has long been known to increase cancer incidence among affected individuals (76). Therefore potential risks to human body after long-term exposure should be taken seriously.

Moreover, the combined effects of exposure multi-metal cannot be ignored. In summary, Cr (VI) and As could be considered key carcinogenic risk factors affecting the health of local residents, which was consistent with previous observations in Suzhou (34) and Zhejiang Province (59). Hence, it is necessary for the local government to take pertinent actions to control the emission sources of PM_2.5_-bound metals and metalloids.

## Conclusion

This study provides a four-year reference for experimental and field studies on PM_2.5_. The annual average concentration of PM_2.5_ in Wuxi from 2020 to 2023 was 40.42 ± 26.11 μg/m^3^, showing a downward trend over the years. This shows that Wuxi has achieved certain results in environmental protection and energy conservation over the past 4 years. However, although the annual mean concentration of PM_2.5_ in Wuxi met the CNAAQS IT-2 standard, there was still a gap compared to CNAAQS IT-1 and WHO standards, suggesting that the energy conservation and emission reduction should not be overlooked. During the monitoring period, the concentrations of PM_2.5_ indicated clear seasonal distribution characteristics, with levels in winter and spring significantly higher than those in summer and autumn.

Fe, Al, Zn, Mn, Pb, Cu, and Ba were seven dominant metals in PM_2.5_ accounted for 95.7% of TMs. During the monitoring period over the past 4 years, the concentrations of most metals in PM_2.5_ showed a remarkable decline, as Co showed the largest reduction in concentration, dropping by 55.1%, followed by Sr. and Cu. Seasonal variation could observed in Pb, Zn, Ba, Ag, Rb, Al, Cd, Sb, Tl, Sr., Mn, Ni, and As, with higher concentration in winter.

PMF analysis revealed that metals in PM_2.5_ in Wuxi City mainly generated from fossil fuels combustion, industrial pollution, vehicle emissions, and construction dust pollution. The sequence of non-carcinogenic metals by their mean HQ values was found to be Mn > As > Pb > Cd > Co > Ni > Al > V > Mo > Sb > Cr (VI) > Zn > Cu > Se. And the sequence of carcinogenic metals by their mean RISK values was found to be As > Cr (VI) > Cd > Ni > Pb, During the monitoring period.

Considering both non-carcinogenic risk and carcinogenic factors, the risk levels of individual elements monitored during the study were within acceptable range according to EPA. However, it is worth noting that PM_2.5_ in the air can enter the human body not only through inhalation but also through both oral and skin exposure. Hence, long-term exposure risks multiple pathways should not be ignored, especially for Mn, which had the highest HQ in the monitored metals.

Moreover, the RISK values of As and Cr (VI) were above 1 × 10^−6^, indicating that the potential health risks from long-term exposure cannot be ignored. It is also noteworthy that even after conversion by 1/7, the concentration of Cr (VI) still exceeded the CNAAQS standard. This highlights the need for the accurate detection of Cr (VI) concentrations to precisely estimate its risk, as it remained the most hazardous metal during the monitoring period.

This study suggests that future efforts should not only continue promoting clean energy, green transportation, civilized construction and decreasing industrial pollution emissions to reduce the PM_2.5_ concentration, but also increase the frequency of air monitoring in winter and spring. Additionally, targeted measures should be taken to reduce the concentrations of Cr (VI), As and Mn, to better protect the health of local residents in Wuxi.

## Data Availability

The raw data supporting the conclusions of this article will be made available by the authors, without undue reservation.
