# Supplementary material for: Environmental surveillance of the health risk of PM2.5-bound metals and metalloids in Wuxi, China, from 2020 to 2023
Source: Front Public Health. 2025 Sep 8;13:1599702. doi: 10.3389/fpubh.2025.1599702 (PMC12450981; doi:10.3389/fpubh.2025.1599702)
Supplement: Supplementary file 1 [file Data_Sheet_1.docx]

**Table S1 RfC and IUR values of PM2.5-bound metals and metalloids via inhalation**

| Metals and metalloids | Non-carcinogen | |  | Carcinogen | |
| --- | --- | --- | --- | --- | --- |
|  | RfC（mg/m^3^） | Datasources |  | IUR（μg/m^3^）^-1^ | Datasources |
| Sb | 3.00E-04 | IRIS |  |  |  |
| Al | 5.00E-03 | PPRTVs |  |  |  |
| As | 1.50E-05 | CALEPA |  | 4.30E-03 | IRIS |
| Cd | 1.00E-05 | IRIS |  | 1.80E-03 | ATSDR |
| Cr^6+^ | 1.00E-04 | IRIS |  | 1.20E-02 | IRIS |
| Pb | 1.50E-04 | NAAQS |  | 1.20E-05 | CALEPA |
| Mn | 5.00E-05 | IRIS |  |  |  |
| Ni | 9.00E-05 | [39] |  | 2.60E-04 | [32] |
| Se | 2.00E-02 | CALEPA |  |  |  |
| Co | 6.00E-06 | PPRTVs |  |  |  |
| Cu | 4.02E-02 | [39] |  |  |  |
| Mo | 4.00E-04 | [39] |  |  |  |
| V | 1.00E-04 | [39] |  |  |  |
| Zn | 3.01E-01 | [39] |  |  |  |
